# Protective effect of rutin in comparison to silymarin against induced hepatotoxicity in rats

**DOI:** 10.14202/vetworld.2017.74-80

**Published:** 2017-01-19

**Authors:** M. Kasi Reddy, A. Gopala Reddy, B. Kala Kumar, D. Madhuri, G. Boobalan, M. Anudeep Reddy

**Affiliations:** 1 Department of Veterinary Pharmacology and Toxicology, P V Narsimha Rao Telangana Veterinary University, College of Veterinary Science, Rajendranagar, Hyderabad - 500 030, Telangana, India; 2 Department of Veterinary Pathology, P V Narsimha Rao Telangana Veterinary University, College of Veterinary Science, Rajendranagar, Hyderabad - 500 030, Telangana, India

**Keywords:** acetaminophen, hepatotoxicity, rutin, silymarin

## Abstract

**Aim:**

The aim of this study is to evaluate the hepatoprotective effect of rutin (RTN) in comparison to silymarin (SLM) against acetaminophen (APAP)-induced hepatotoxicity in rats.

**Materials and Methods:**

Male Wistar albino rats (n=24) of 3 months age were equally divided into four groups. Group 1 served as normal control. Hepatotoxicity was induced in the remaining three groups with administration of 500 mg/kg po APAP from day 1-3. Groups 2, 3, and 4 were subsequently administered orally with distilled water, 25 mg/kg of SLM, and 20 mg/kg of RTN, respectively, for 11 days. The mean body weights and biomarkers of hepatotoxicity were estimated on day 0, 4 (confirmation of toxicity), and 15 (at the end of treatment). Hematological parameters were evaluated on day 4 and 15. Antioxidant profile and adenosine triphosphatases (ATPases) were assessed at the end of the experiment. Liver tissues were subjected to histopathology and transmission electron microscopy after the sacrifice on day 15.

**Results:**

Antioxidant profile, ATPases, and hematological and sero-biochemical parameters were significantly altered, and histopathological changes were noticed in the liver of toxic control group. These changes were reversed in groups 3 and 4 that were administered with SLM and RTN, respectively.

**Conclusion:**

The results of the present investigation enunciated that SLM has potent hepatoprotective activity though the RTN was found superior in restoring the pathological alterations in paracetamol-induced hepatotoxicity in Wistar albino rats.

## Introduction

Drug-induced hepatotoxicity accounts for the withdrawal of substantial amounts of clinically approved drugs from the market [1]. Drug-induced hepatotoxicity mimics both acute and chronic forms of liver disease [2]. Most of the drugs are metabolized in liver, and factors such as alcohol consumption, malnutrition, infection, and anemia affect the organ’s functional activity causing drug-induced injury [3].

Acetaminophen (N-acetyl-p-aminophenol/APAP; paracetamol [PCM]) is a widely used over-the-counter antipyretic/analgesic agent [4,5], which is bio-activated to a reactive metabolite (N-acetyl-p-benzoquinone imine) in the liver that binds with cellular proteins covalently, deplete hepatic glutathione, and substantially cause fatal centrilobular hepatic necrosis in a dose-dependent fashion [6]. Drug-induced acute liver injury due to APAP overdose is the most frequent cause and used as a reliable model by clinical researchers for assessing the hepatoprotective effect of novel compounds [7,8].

Silymarin (SLM) is a lipophilic extract isolated from the seeds and fruits of *Silybum marianum* (milk thistle), is the folklore and thoroughly investigated plant in the curing of liver ailments [9]. SLM offered protection against chemical hepatotoxins such as ethanol, CCl_4_, and APAP [10]. SLM is an effective treatment for reducing hepatic steatosis in patients with non-alcoholic fatty liver disease [11]. It has various clinical applications, namely, toxic and drug-induced liver diseases, alcoholic liver diseases, diabetic patients, liver cirrhosis, viral hepatitis, and mushroom poisoning [12].

Rutin (RTN) is a natural glycoside between the flavonol quercetin and disaccharide rutinose (α-L-Rhamnopyranosyl-(1,6)-β-D-glucopyranose). In humans, RTN binds to Fe^2+^, thereby preventing it from binding to hydrogen peroxide, and inhibits free radical-induced cellular damage, thus acting as an antioxidant [13]. Furthermore, RTN has been shown to inhibit the vascular endothelial growth factor in subtoxic concentrations *in vitro*, and hence acts as an inhibitor of angiogenesis [14].

Hence, an experimental study was conducted to assess the hepatoprotective effect of RTN and to compare its efficacy with SLM against APAP-induced hepatotoxicity in male Wistar albino rats.

## Materials and Methods

### Ethical approval

The experimental protocol was approved by the Institutional Animal Ethics Committee (Approval No. CPCSEA I/09/2014, Dated: 27.11.2014).

### Chemicals

All the chemicals used in the biochemical analysis were of analytical grade and obtained from Qualigens Pharma Pvt. Ltd., Mumbai, Himedia Laboratories Pvt. Ltd., Mumbai, and Sisco Research Laboratories Pvt. Ltd., Mumbai, India.

### Animals

Male Wistar albino rats (n=24) of 3 months old were procured from Sanzyme Pvt. Ltd., Hyderabad, India. All the animals were maintained under standard conditions prescribed by IAEC.

### Induction of experimental hepatotoxicity

Following 24 h fasting with free access to water *ad-libitum*, hepatotoxicity was induced in groups 2, 3, and 4 with 500 mg/kg of APAP per oral administration for 3 consecutive days [15].

### Experimental design

The experimental study was carried out on 24 rats that were randomly divided into four groups comprising 6 rats in each group.

Group 1: Normal control.

Group 2: APAP (500 mg/kg po once daily for 3 days) and distilled water (5 ml/kg po once daily) were administered subsequently for 11 days since the last dose of APAP.

Group 3: APAP (as in Group 2) and 25 mg/kg po of SLM once daily were administered subsequently for 11 days since the last dose of APAP.

Group 4: APAP (as in Group 2) and 20 mg/kg po of RTN once daily were administered subsequently for 11 days since the last dose of APAP.

The mean body weights of the groups were estimated on day 0, 4 (confirmation of toxicity), and 15 (at the end of treatment). Hematological parameters (total erythrocyte count [TEC], total leukocyte count, hemoglobin [Hb], packed cell volume [PCV], and prothrombin time [PT]) were estimated in all the groups on day 4 and 15. On the 15^th^ day, blood samples were collected and centrifuged; sera were separated and stored at −80°C until assayed for alanine transaminase, bilirubin, total protein, and glucose. At the end of the experiment, liver tissues were collected for the assay of reduced glutathione (GSH), glutathione peroxidase (GPx), superoxide dismutase (SOD), catalase (CAT), and thiobarbituric acid reactive substances (TBARS). The liver tissue was collected in ice-cold conditions for the enzyme assays such as adenosine triphosphatases (ATPases). Histopathological and transmission electron microscopic (TEM) studies were conducted on liver tissues to draw possible conclusions at the end of the experiment.

### Estimation of antioxidant and peroxidation parameters

Liver samples were homogenized in 1 ml of 10 mmol/l Tris–HCl buffer of pH 7.1 and centrifuged at 12,000 g for 10 min. The supernatant was separated for the estimation of enzymatic and non-enzymatic antioxidants levels, namely, SOD [16], CAT [17], GSH [18], GPx [19], and TBARS [20].

### Estimation of liver function biomarkers

Hepatic alanine transaminase, total bilirubin, glucose, and total protein concentration in serum were estimated as per the instructions given in the Erba Diagnostics kits.

### Histopathology

At the end of the study, all the animals were sacrificed; liver samples were collected and fixed in 10% neutral buffered formalin. Subsequently, the fixed samples were processed and stained with Hematoxylin and Eosin stain as described by Singh and Sulochana [21].

### TEM

For microscopic studies, the samples were transferred to vials and fixed in 3% glutaraldehyde, stained with saturated aqueous uranyl acetate and counterstained with 4% lead citrate [22] and were observed at different magnifications under TEM (Model: Hitachi, H-7500) at RUSKA Lab, College of veterinary Science, Hyderabad, India.

### Statistical analysis

The experimental data were analyzed by one-way ANOVA using Statistical Package for Social Sciences version 21.0. The mean difference was estimated using Duncan’s multiple range test (p<0.05) [23].

## Results

### Body weights

Mean body weights of APAP control group were non-significantly decreased as compared to day 0 value on day 4 and 15. The body weights were non-significantly increased in the groups 1, 3, and 4 at the end of the 15^th^ day as compared to their respective day 0 values (Table-1).

**Table-1:** Mean body weights (g) of different groups of rats.

Group	Control	APAP control	APAP+SLM	APAP+RTN
Day 0	214.60±5.38	234.83±11.71	242.83±10.87	209.17±12.27
Day 4	221.40±5.67	229.67±12.29	240.00±10.70	204.50±12.12
Day 15	230.00±7.70	207.50±17.01	253.83±10.47	216.33±12.24

Values are mean±SE (n=6); one-way ANOVA (SPSS). APAP=Acetaminophen, SLM=Silymarin, RTN=Rutin, SE=Standard error, SPSS=Statistical Package for Social Sciences

### Liver functional biomarkers

Groups 2, 3, and 4 showed significantly (p<0.05) elevated levels of alanine aminotransferase (ALT) and glucose and total bilirubin, and reduced concentration of total protein as compared to the control group 1 on day 4. The above-altered liver biomarkers were significantly (p<0.05) reversed in groups 3 and 4 in comparison to group 2 and their respective day 4 values (Table-2).

**Table-2:** Liver functional biomarkers in different groups of rats.

Group	Control	APAP control	APAP+SLM	APAP+RTN
Total protein concentration (g/dl)				
Day 4	8.41±0.18	4.83±0.50*	4.83±0.56*	4.81±0.65
Day 15	8.43±0.59	4.81±0.78*	7.76±0.72	7.28±0.73
Total bilirubin concentration (mg/dl)				
Day 4	0.75±0.01	1.38±0.01*	1.36±0.00*	1.35±0.01*
Day 15	0.83±0.00	1.37±0.00*	0.85±0.01	0.89±0.01
Glucose concentration (g/dl)				
Day 4	82.28±0.58	117.38±0.77*	115.08±0.77*	116.39±0.73*
Day 15	85.19±1.00	111.78±3.70**	96.85±1.10*	98.74±0.52*
ALT activity (IU/L)				
Day 4	37.27±0.63	92.16±0.59*	89.06±0.47*	90.12±0.55*
Day 15	36.32±0.73	93.24±0.67**	58.38±0.63*	62.64±0.48*

Values are mean±SE (n=6); one-way ANOVA (SPSS),

* p<0.05,

** p<0.01 in relation to group 1 (Duncan’s multiple comparison test). APAP=Acetaminophen, SLM=Silymarin, RTN=Rutin, ALT=Alanine aminotransferase, SE=Standard error, SPSS=Statistical Package for Social Sciences

### Anti-oxidation and peroxidation parameters

Acute APAP treatment significantly (p<0.05) decreased the levels of antioxidants in the liver tissue (e.g. SOD, CAT, and GSH) whereas the level of peroxidation markers such as TBARS and GPx was significantly (p<0.05) increased compared to the control group. Administration of SLM and RTN in groups 3 and 4 showed significant (p<0.05) reversal of the above values as compared to the APAP-induced hepatotoxic group 2 (Table-3).

**Table-3:** Antioxidant and peroxidation parameters of liver in different groups of rats.

Group	Control	APAP control	APAP+SLM	APAP+RTN
TBARS concentration (n moles of MDA released/mg protein)	18.50±0.32	23.19±0.48*	19.60±0.29*	20.18±0.34*
GSH concentration (n moles/mg protein)	7.52±0.16	3.68±0.16*	5.56±0.17*	5.86±0.11*
GP_X_ activity (U/mg protein)	20.6±0.27	17.38±0.27*	19.78±0.28	18.82±0.33*
SOD activity (U/mg protein)	10.83±0.23	7.23±0.19*	8.23±0.16*	8.16±0.15*
Catalase activity (U/mg protein)	4.94±0.01	3.18±0.01*	4.64±0.02*	4.19±0.0*

Values are mean±SE (n=6); one-way ANOVA (SPSS),

* p<0.05 in relation to group 1 (Duncan’s multiple comparison test). APAP=Acetaminophen, SLM=Silymarin, RTN=Rutin, TBARS=Thiobarbituric acid reactive substances, MDA=Malondialdehyde, GSH=Reduced glutathione, GPx=Glutathione peroxidase, SOD=Super oxide dismutase, SE=Standard error, SPSS=Statistical Package for Social Sciences

### Membrane bound enzymes

The activity of Mg^2+^ATPase and Na^+^/K^+^ ATPase in the liver tissue was significantly (p<0.05) increased in groups 3 and 4 when compared to APAP-induced hepatotoxic control group. However, there was no significant variation in the activity among therapeutic groups as compared to group 1 (Table-4).

**Table-4:** ATPase (μg of Pi liberated/mg microsomal protein/30 min) of liver in different group of rats.

Group	Control	APAP control	APAP+SLM	APAP+RTN
Mg^+2^ ATPase activity	4.36±0.14	2.62±0.11*	3.70±0.07*	3.63±0.08*
Na^+^K^+^ATPase activity	7.49±0.14	6.28±0.11*	7.36±0.15	7.08±0.15

Values are mean±SE (n=6); one-way ANOVA (SPSS),

* p<0.05 in relation to group 1 (Duncan’s multiple comparison test). APAP=Acetaminophen, SLM=Silymarin, RTN=Rutin, ATPase: Adenosine triphosphatase, SE=Standard error, SPSS=Statistical Package for Social Sciences

### Hematological parameters

Acute APAP treatment significantly (p<0.05) decreased the levels of the TEC, total leukocyte count, Hb, and PCV, while significantly (p<0.05) increased the PT in APAP-induced hepatotoxic group. Administration of SLM and RTN in the treatment groups showed significant (p<0.05) reversal of the above values as compared to the APAP-treated group alone (Table-5).

**Table-5:** Hematological parameters of different group of rats.

Group	Control	APAP control	APAP+SLM	APAP+RTN
Total erythrocyte count (10^6^/mm^3^)				
Day 4	8.34±0.16	6.32±0.09*	6.34±0.12*	6.43±0.09*
Day 15	8.46±0.18	6.23±0.16*	8.13±0.13	7.84±0.10*
Total leukocyte count (10^3^/mm^3^)				
Day 4	8.63±0.11	10.43±0.12*	10.24±0.11*	10.36±0.11*
Day 15	8.74±0.11	10.51±0.11*	8.82±0.09	8.94±0.09
Hemoglobin concentration (g/dl)				
Day 4	16.56±0.17	12.32±0.23*	12.38±0.23*	12.48±0.18*
Day 15	16.42±0.19	12.53±0.19*	16.14±0.24	15.72±0.17*
Packed cell volume (%)				
Day 4	42.64±0.27	34.36±0.18*	33.86±0.15*	34.57±0.12*
Day 15	43.14±0.22	34.32±0.09*	41.43±0.09*	40.91±0.05*
Prothrombin time (s)				
Day 4	14.88±0.57	26.32±0.59*	26.16±0.79*	26.28±0.84*
Day 15	14.88±0.57	26.32±0.59*	16.47±0.67	17.43±0.54*

Values are mean±SE (n=6); one-way ANOVA (SPSS),

* p<0.05 in relation to group 1 (Duncan’s multiple comparison test). APAP=Acetaminophen, SLM=Silymarin, RTN=Rutin, ATPase: Adenosine triphosphatase, SE=Standard error, SPSS=Statistical Package for Social Sciences

### Histopathology

**Figure-1:**
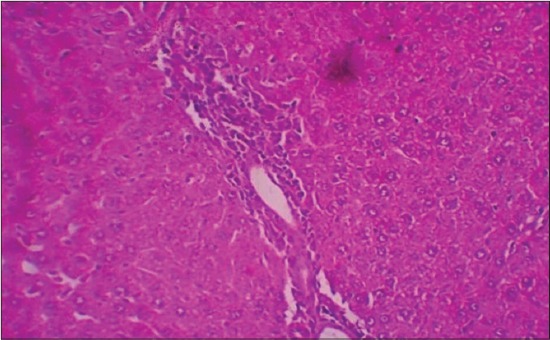
Photomicrograph of liver showing sinusoidal dilatation and periportal infiltration (H and E, ×200 - Group 2).

**Figure-2:**
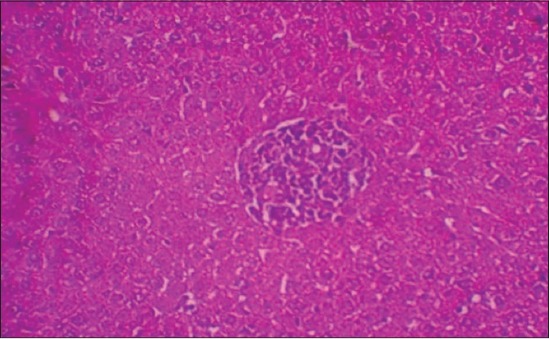
Photomicrograph of liver showing mononuclear infiltration and few cells of coagulative necrosis (H and E, ×200 - Group 2).

**Figure-3:**
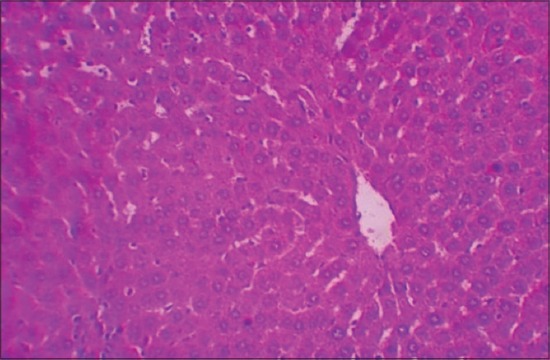
Photomicrograph of liver showing mild sinusoidal dilatation and regenerative changes (H and E, ×200 - Group 3).

**Figure-4:**
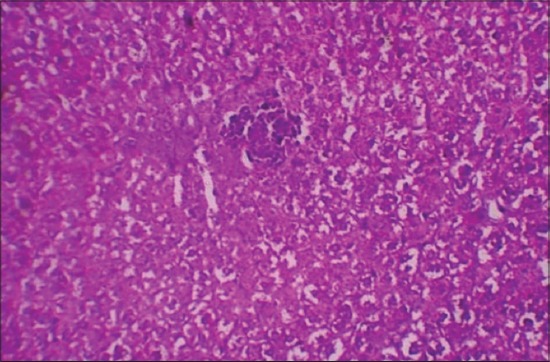
Photomicrograph of liver showing moderate degenerative changes and mononuclear infiltration (H and E, ×200 - Group 4).

The histological examination of liver sections from APAP toxic control (group 2) revealed sinusoidal dilatation and periportal infiltration (Figure-1), coagulative necrosis, and mononuclear infiltration (Figure-2). Group treated with SLM (group 3) showed mild sinusoidal dilatation and regenerative changes and RTN-treated group (group 4) revealed mild congestion, mononuclear infiltration, and moderate degenerative changes (Figures-3 and 4).

### TEM

**Figure-5:**
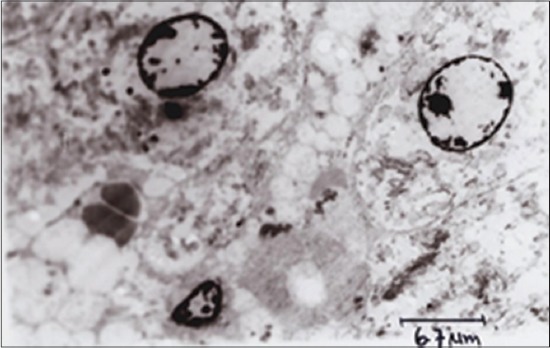
Transmission electron microscopy of hepatocyte showing altered architecture, i.e., distorted mitochondria and rough endoplasmic reticulum, eccentric nucleus and pyknotic, chromatin margination, Kupffer cells, and mild fatty changes in cytoplasm (Group 2).

**Figure-6:**
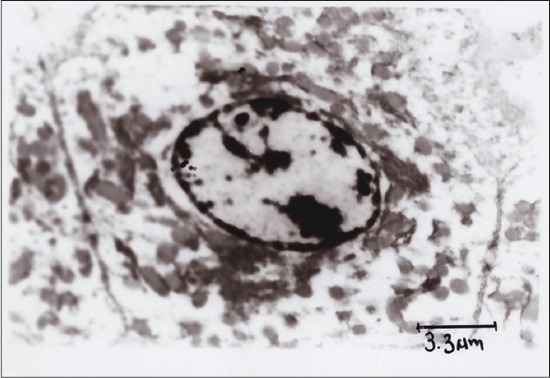
Transmission electron microscopy of hepatocyte showing regenerated mitochondria, rough endoplasmic reticulum, normal nucleus, nucleolus and cytoplasm showing vesicular structures (Group 3).

**Figure-7:**
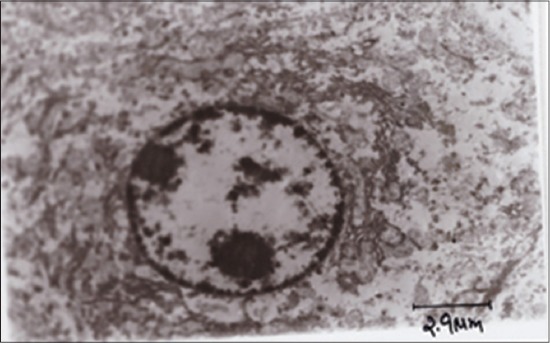
Transmission electron microscopy of hepatocyte showing uniform mitochondrial distribution, regeneration of rough endoplasmic reticulum and vacuolations in cytoplasm (Group 4).

TEM of hepatocyte of APAP toxic group 2 (Figure-5) showed shrunken hepatocytes, margination of chromatin, fatty degeneration, Kupffer cells, and clumping of mitochondria. SLM-treated group (Figure-6) showed mild margination of chromatin, condensed cytoplasm, and regeneration of RER, while the RTN-treated group (Figure-7) showed uniform mitochondrial distribution, regeneration of rough ER, and vacuolations in cytoplasm.

## Discussion

APAP is known to cause hepatic damage at very high doses and hence used as an efficient model to assess the efficacy of novel hepatoprotective agents [15]. N-acetyl-p-benzoquinone imine is the toxic metabolite of APAP, which induces lipid peroxidation and cell necrosis by covalently binding to the sulfhydryl groups of proteins [4]. The membrane-damaging effects of N-acetyl-p-benzoquinone-imine result in the leakage of the hepatocytic contents in liver injury caused by APAP overdose [24], and hence there is an increase in serum enzyme levels. The involvement of oxidative stress in APAP-induced hepatotoxicity creates the need for searching of a novel compound with potent antioxidant and protective activity.

The reductions of mean body weights in APAP control group are mainly due to oxidative stress and reactive oxygen species generated in APAP-induced hepatotoxicity and altered metabolism of liver [25]. Weight reduction may also be attributed to hyperglycemia induced in this study, which adds to the catabolism of proteins, resulting in weight loss. Weight gain observed in the animals treated with SLM and RTN (groups 3 and 4, respectively) may be due to restoration of antioxidant defenses and recovery of hepatic histoarchitecture as evident from the findings of this study.

The normal levels of serum markers such as ALT, total bilirubin, and glucose were increased and total proteins were significantly decreased in this study which could be due to loss of functional integrity of cell membranes in the liver. This is in accordance with the finding of Kiran *et al*. [26]. Treatment with SLM and RTN decreased the activity of the above marker enzyme on day 15, suggesting their protective effect in this study. These observations support that the regeneration of hepatocytes and healing of hepatic parenchyma reverse the serum transaminases levels to normal [27].

In the current investigation, the GSH level of liver tissue was improved in the groups treated with SLM and RTN when compared to toxic group 2. However, SLM surpassed RTN extract, which might be due to the ability of SLM to generate GSH and maintain GSH homeostasis in the body [27]. Increased levels of malondialdehyde in toxic control group signify that APAP triggers massive lipid peroxidation [4,28], which is responsible for liver injury. RTN showed free radical scavenging ability that was comparable to SLM. The activities of SOD and CAT were significantly increased in the SLM- and RTN-treated groups as compared to toxic group 2 and also there was a corresponding decrease in TBARS (extent of lipid peroxidation) in the liver of treated groups 3 and 4.

The activities of Na^+^/K^+^ ATPase and Mg^2+^ ATPase in liver were significantly increased in the treatment groups, which might be due to the membrane stabilizing activity of SLM and RTN owing to their antioxidant potential. This may be attributed to the changes in the levels of total cholesterol and triglycerides as the activity of ATPases is more likely to be changed with the change in the level of these lipids [29].

The TEC, Hb, and PCV in APAP control group 2 were significantly lowered as compared to all the other groups at respective time intervals from 4^th^ to 15^th^ day. It may due to hepatic injury induced by APAP [24]. Findings of our study further revealed that both SLM and RTN reversed these alterations in the hematological parameters. The reversal of red blood cell counts and Hb levels observed in case of SLM- and RTN-treated groups could be attributed to the protective effects on tissue repair and deceleration of disease progression. Further, a significant rise in white blood cell count in PCM control group in this study is possibly due to the stimulation of immune system against the invading antigens and also to an interleukin-1β mediated rise in the respective colony-stimulating factors.

In the present study, APAP-induced PT prolongation seen in the APAP control group 2 may be attributed to hepatic injury resulting in the reduced production and activation of certain blood clotting factors as the metabolic machinery is compromised in the liver [30]. The treatment improved the clotting time owing to the regeneration of hepatic histoarchitecture and its physiology.

Further, the histopathology and TEM of liver in toxic control group revealed altered histoarchitecture, but regenerative changes were noticed in groups treated with SLM and RTN, thus exhibiting their potent hepatoprotective action by stabilizing cellular plasma membrane.

In conclusion, the results of the present investigation enunciated that RTN could improve hepatic function by regenerating the histoarchitecture and functioning of liver in APAP-induced hepatic damage, which was evident from sero-biochemical and liver tissue oxidant-antioxidant biomarker parameters, and histopathology. The results of the group treated with RTN were comparable to that of SLM-treated group. The beneficial effects of RTN are attributed to the antioxidant and free radical scavenging properties.

## Authors’ Contributions

This study was a part of MKR’s research work during his M.V.Sc. program. AGR, BKK, and DM designed the experiment. MKR carried out the experiment under the guidance of AGR, BKK, and DM. GB and MAR assisted in laboratory works and drafting the manuscript. All authors have read and approved the final manuscript.
